# Challenges in Optimizing Nanoplatforms Used for Local and Systemic Delivery in the Oral Cavity

**DOI:** 10.3390/pharmaceutics16050626

**Published:** 2024-05-07

**Authors:** Dorin Ioan Cocoș, Olimpia Dumitriu Buzia, Alin Laurențiu Tatu, Monica Dinu, Lawrence Chukwudi Nwabudike, Claudia Simona Stefan, Kamel Earar, Carmen Galea

**Affiliations:** 1 Centre in the Medical-Pharmaceutical Field, Faculty of Medicine and Pharmacy, “Dunarea de Jos” the University of Galati, 800008 Galati, Romania; cdorin1123@gmail.com (D.I.C.); claudia.stefan@ugal.ro (C.S.S.); erar_dr.kamel@yahoo.com (K.E.); 2 Clinical Medical Department, Faculty of Medicine and Pharmacy, “Dunarea de Jos” University, 800008 Galati, Romania; dralin_tatu@yahoo.com; 3 Dermatology Department, “Sf. Cuvioasa Parascheva” Clinical Hospital of Infectious Diseases, 800179 Galati, Romania; 4 Multidisciplinary Integrative Center for Dermatologic Interface Research MIC-DIR, 800010 Galati, Romania; 5 N. Paulescu Institute, 030167 Bucharest, Romania; chukwudi.nwabudike@live.com; 6 Department of Medical Disciplines, Faculty of Dental Medicine, University of Targu Mures, 540099 Targu Mures, Romania; unicapharm@gmail.com

**Keywords:** nanoplatforms, nanocarrier, transdermal drug delivery, microneedles, nanoparticles, oral pathology

## Abstract

In this study, we focused on innovative approaches to improve drug administration in oral pathology, especially by transmucosal and transdermal pathways. These improvements refer to the type of microneedles used (proposing needles in the saw), to the use of certain enhancers such as essential oils (which, besides the amplifier action, also have intrinsic actions on oral health), to associations of active substances with synergistic action, as well as the use of copolymeric membranes, cemented directly on the tooth. We also propose a review of the principles of release at the level of the oral mucosa and of the main release systems used in oral pathology. Controlled failure systems applicable in oral pathology include the following: fast dissolving films, mucoadhesive tablets, hydrogels, intraoral mucoadhesive films, composite wafers, and smart drugs. The novelty elements brought by this paper refer to the possibilities of optimizing the localized drug delivery system in osteoarthritis of the temporomandibular joint, neuropathic pain, oral cancer, periodontitis, and pericoronitis, as well as in maintaining oral health. We would like to mention the possibility of incorporating natural products into the controlled failure systems used in oral pathology, paying special attention to essential oils.

## 1. Introduction

The use of transdermal systems in dentistry aims to release active substances directly at the site of action, accumulating in the targeted tissue and providing an increase in the stability of the active substances. In 1906, Erlich was the first to introduce the term “magic bullet”, referring to systems with controlled failure [1]. Transdermal systems appeared in 1997, and the first substance released using the transdermal system was rituximab, approved by the FDA in the same year [2]. 

Transmucosal systems will become preferred pathways, especially in antitumor research because they provide targeted therapy at the site of action, using the minimum dose required through metabolic mechanisms that are mediated by ligands. Transdermal and transmucosal systems aim to deliver active substances either to the gastrointestinal tract or to other more distant tissues. For controlled yielding oral administration to achieve its goal, there are several conditions. It is very important in this respect that the active substances remain intact after passing through the gastrointestinal absorbent epithelium, respectively after hepatic capture. Controlled release systems should resist biological digestion and avoid the first-pass effect [1,2]. The second type of transdermal and transmucosal systems allows a destructuring of active substances at the gastrointestinal level followed by self-assembly. It represents a process of spontaneous aggregation of molecules into a stable nanostructure due to electrostatic interaction, van der Waals bonds, and hydrogen bonds [3,4].

### Principles of Release of Active Substances into the Oral Cavity 

In the case of transmucosal administration, the active substances are released into the oral cavity to be absorbed by the oral mucosa. Numerous studies have considered the use of oral cavity peculiarities to deliver various active substances both locally and systemically. In transmucosal delivery, medicinal substances are not exposed to the action of aggressive acids and enzymes encountered along the digestive tract. On the other hand, the first hepatic passage is bypassed and is a good option for patients who cannot receive enteral drugs [5,6].

The drugs cross the squamous epithelium of the oral cavity through two pathways: the transcellular and paracellular pathways. The transcellular pathway is suitable for lipophilic drugs that cross the cell membrane to enter the cell and then leave the cell to enter another cell [7,8,9]. Hydrophilic drugs, conversely, are absorbed through junctions between cells into intracellular spaces. These drugs lend themselves to the paracellular pathway [10,11].

The distribution of active substances in the oral cavity can be divided into distribution-generating systemic effects or local effects. The active substances released in the oral cavity can address soft and hard tissues. This study takes into account the fact that the administration of active substances in the oral cavity for local effect is too little used, and we try to propose various active substances that are suitable for controlled failure by controlled transdermal and transmucosal delivery systems [1,2,12]. Administration in the form of tablets, or capsules (operculate or gelatinous), remains an inconvenient method for many categories of patients (elderly, children, psychiatric patients, patients under anesthesia, patients with swallowing problems, etc.). In addition, several aggregation excipients and flavoring agents are added to these pharmaceutical forms, the effects of which may be unforeseeable. All these aspects motivate interdisciplinary teams consisting of doctors, pharmacists, biochemists, and engineers to pay more attention to intelligent intraoral release systems (SIS) [1,2,12].

To increase penetration into the oral cavity, penetration amplifiers are used, which increase the fluidity of cell membranes and drug permeability. The most well-known penetration enhancers are surfactants, chelating agents, fatty acids, and alcohols [13,14]. To the aforementioned penetration enhancers are added essential oils for which comparative evaluation studies have been made, demonstrating the role of penetration enhancers in transdermal drug delivery [15].

In dentistry, the most used controlled failure form refers to patches, due to their rapid onset time and sustained drug release. The oral cavity is an ideal area of application, which is also due to the immobility of tissues at this level [16]. Although there are limitations related to the limited surface of the oral cavity and the action of saliva, they can be removed using effective agents and choosing a correct method of administration. The use of dissolvable microneedles will allow dental teams a more comfortable administration of local anesthetics, without the inconvenience caused by local injection currently practiced [17].

Consulting the specialized literature, we found that pre-existing studies usually focus on a certain pharmaceutical form with controlled release to address oral pathology such as mucoadhesive patches [16], microneedles [17,18], microcontainers [19], wafers [20], and chewing gums [21]. The second approach model refers to the use of transmucosal and transdermal controlled release systems used in neuropathic pain [22,23] and in trigeminal neuralgia [24], and of the transmucosal system in the treatment of periodontal pockets [25], in periodontal regeneration [26], and in oral cancer [27,28,29]. The third type of reporting on the transdermal and transmucosal systems used in oral pathology are particularly focused on the various possibilities of improving absorption, whether we refer to it as a question of excipients with an amplifying effect [15,28,30] or to that of physical amplifiers (microneedles, iontophoresis, sonophoresis, and electrophoresis) [31,32,33,34]. Unlike the previously mentioned works, our work presents a more complex approach, considering a varied oral pathology as well as various types of controlled release systems that address these pathologies. The emphasis is, however, on the optimization possibilities potential of these systems as well as on the justification of the solutions proposed by us and their graphic illustration.

Although there are studies on local and/or systemic release in the oral cavity, we have not found any that offer solutions for such diverse pathologies of the oral cavity, generally utilizing controlled release on a particular oral disease. Our main motivation was to provide insights into innovative techniques for administering drugs for oral pathology with a focus on innovation, efficiency, and oral health benefits. The systems proposed by us address various oral pathologies: osteoarthritis of the temporomandibular joint, oral cancer, periodontitis, pericoronitis, and dental caries. In this regard, we proposed using microneedles in the saw to obtain a more efficient delivery than using standardized microneedles, as well as the use at alternating heights of these microneedles in the saw. On the other hand, we suggested that it is of interest to use nanoparticles of essential oils both for their role in maintaining oral health and for their enhancing action. We also envisioned a system of copolymer membranes cemented directly onto the tooth for targeted and sustained drug delivery. An important aspect of our paper refers to highlighting the systems proposed by us and translating them into suggestive images, which provides a clearer image of the proposed optimization systems.

## 2. Physiology of Oral Cavity Pathology 

The oral cavity has several functional structures, including the tongue, salivary glands, cheeks, lips, and palate including teeth, all of which play an important role in physiological processes [35,36]. Histologically, the oral mucosa is a multilayered structure consisting of the *epithelium* (basement membrane and chorion), and the epithelium presents the *stratum basale* (basal layer), *stratum spinosum* (spinous layer), *stratum granulosum* (granular layer), and *stratum corneum* [35,36,37,38]:

- The *stratum basale*, also called the *stratum germinativum*, is the deepest and is arranged on the basement membrane. This layer consists of two tall rows of cells with cuboidal or prismatic shape. These cells have intensely colored voluminous nuclei located in the basal third of the cells. Basal cells exhibit intense metabolism and frequent mitoses that provide flaking turnovers [35,36,37,38];
- The *stratum spinosum* is arranged above the basal layer and presents 7–8 rows of well-defined polyhedral cells with visible intercellular spaces; between the cells of the *stratum spinosum* there are fine cytoplasmic filaments arranged in the form of thorns. The nuclei of the cells of the *spinosum stratum* stain less intensely than the nuclei of the cells of the *stratum basale*, so that the *stratum spinosum* is well delineated from the *stratum basale*. The cells in the deep areas of the *stratum spinosum* show mitosis and together with the basal cells form the germinative zone of the *epithelium*, and towards the surface, the cells of the *stratum spinosum* become flatter [35,36,37,38];
- The *stratum granulosum* occurs when there is also the *stratum corneum*, and in the absence of the *stratum corneum*, may be absent. It is located above the *stratum spinosum* and consists of 2–3 rows of flattened cells that present kerato-hyaline granules in the cytoplasm [35,36,37,38];
- The *stratum corneum*, also called the keratinized layer, is not always present in the mucosa and has an unstructured appearance composed of overlapping layers of keratin. The cells of the *stratum corneum* are flattened and degenerated with nuclear pycnosis, or without nuclei, they are weakly joined to each other. The epithelium is separated from the dermis by the basement membrane, which presents itself as a condensation of the fundamental substance of the underlying connective tissue [35,36,37,38].

**Figure 1:**
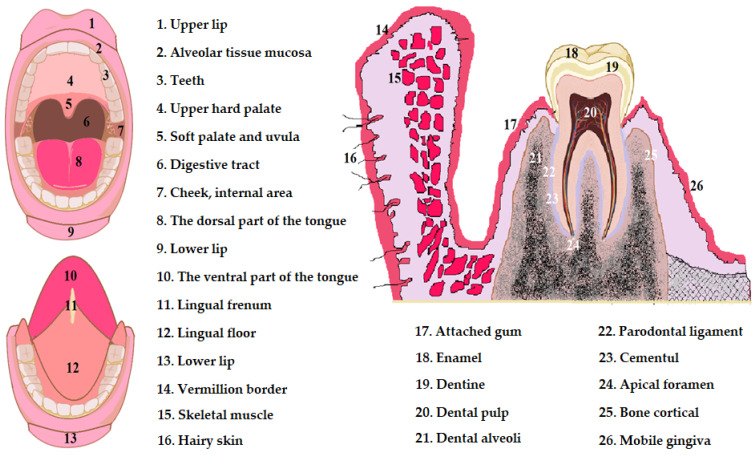
Anatomical description of the oral cavity [35,36,37,38].

The basement membrane is composed of the lamina lucida and lamina densa. The lamina lucida is located towards the basal cells of the epithelium and presents an amorphous structure. The lamina densa is located towards the chorion and has a fibrillar network that consists of reticulin fibers and collagen fibers. The basement membrane continues with those in the dermis or is anchored to them, joining the connective tissue with the basement membrane (Figure 1) [35,36,37,38].

The chorion comprises connective tissue with the role of fixing the epithelium to the deep muscular or bony planes, formed by the superficial dermis located towards the epithelium and the deep dermis. The connective tissue of the dermis is composed of collagen fibers, fibroblasts, and reticulin fibers. The superficial dermis comprises connective tissue rich in cells, and the deep dermis consists of connective tissue containing reticulin and collagen fibers. However, it is 100–200 μm thick compared to the oral mucosa (600 μm). At the level of the dermis, fibroblasts, macrophages, T and B lymphocytes, melanocytes, plasmocytes, and mast cells are identified. The dermis is intensely vascularized, contributing to the red coloration of the mucosa. The vascularization of the papillae of the superficial dermis is terminal and each papilla has a connective tissue–vascular axis made up of lymphatic vessels, arterioles, capillary network, venules, and muscle fibers. The epithelium connective tissue junction located between the epithelium and the dermis is formed by the wavy surface resulting from interpapillary extensions [35,36,37,38].

Gum tissues are keratinized tissues with a thickness of 200 μm and 250 μm, respectively. They have a lower permeability than sublingual and buccal tissue. The gum consists of connective vascular tissue covered by a para-keratinized epithelium and has a firm consistency, becoming looser towards the free margin and presenting interdental papillae in the interproximal spaces [37,38].

Oral saliva is a complex fluid and is essential for maintaining oral health and facilitating initial digestive processes. Saliva also plays an essential role in the transmucosal delivery of drugs, humidifies the oral mucosa and maintains pH. It contains a series of enzymes (phosphatases, esterases, glycosylases), which contribute to the degradation of various drug formulations [39,40,41,42,43].

Oral pathology is a branch of medicine that deals with the study and diagnosis of diseases affecting the temporomandibular joint, oral cavity, lips, tongue, palate, and other oral structures. These conditions may include infections, inflammation, precancerous or cancerous lesions, disorders of the oral mucosa, congenital or acquired abnormalities, diseases of the teeth and gums, and other problems related to the oral cavity [44,45,46,47].

Neuropathic pain is a disorder of varying intensities generated by damage to the central or peripheral nervous system. This category includes trigeminal neuralgia, postherpetic neuralgia, post-traumatic neuropathy, and neuropathies caused by systemic diseases (cancer, diabetes, HIV/AIDS) and drugs [22,48]. Trigeminal neuralgia can mimic the pain of dental etiology, with many patients indicating an intraoral area as the trigger area. Although the treatment of the first choice is carbamazepine, several topical medications (capsaicin, tricyclic antidepressants, monoamine oxidase inhibitors, serotonin reuptake inhibitors) can be applied to the trigger area. Trigeminal neuralgia is a short-term pain affecting several branches of the fifth cranial nerve (trigeminal). The pain is so clearly described that most of the time the diagnosis can be established based on a simple history. With the intraoral area as a triggering area, some patients may confuse it with dental pain [49,50].

Osteoarthritis of the temporomandibular joint is a condition in which the cartilage covering the joint between the lower jaw and the temporal bone at the base of the skull gradually deteriorates. Many patients suffering from osteoarthritis of the temporomandibular joint present with joint pain, stiffness, disturbance of eating, speaking, laughter, and other symptoms in the ear. The causes of temporomandibular joint osteoarthritis are diverse; they may include genetic factors, previous trauma to the joint, lifestyle habits, and general health, but also environmental factors can play an important role in the development and progression of this condition [51,52].

Oral cancer refers to any type of cancer that develops in the oral cavity or oropharyngeal area, which includes the lips, tongue, lining of the cheeks, gums, soft palate, tonsils, and the back of the throat. The most common type of oral cancer is squamous cell carcinoma, which commonly occurs among transplant patients. Although advances have been made in surgery, radiotherapy, and chemotherapy, the prognosis for this diagnosis has not improved considerably over the past 50 years. To enhance the effect of immune agents, extended-release systems can be used to directly target cancer cells, allowing better intracellular penetration and increasing antigen immunity [53,54,55].

Periodontal pockets are abnormal spaces that form between teeth and gums as a result of periodontal disease. Periodontal disease is an inflammatory condition of the tissues that support the teeth, including the gums, alveolar bone, and periodontal ligaments. These pouches form when the dental gingival junction retracts or peels off the teeth due to inflammation and destruction of the supporting tissue. Periodontal pockets are a sign of periodontal disease progression and may be associated with symptoms such as gum bleeding, gum swelling, tooth mobility, and increased sensitivity to heat or cold. Microorganisms that can be found in periodontal pockets include a variety of anaerobic and aerobic bacteria, such as *Prevotella intermedia*, *Porphyromonas gingivalis*, *Fusobacterium nucleatum*, *Actinomyces*, *Bacteroides*, *Treponema*, and others. These bacteria can produce toxic substances that aggravate inflammation and destroy the supporting tissues of teeth [25,56].

Pericoronitis is an inflammatory gum disease due to an incomplete, partial rash or abnormal wisdom tooth eruption. It occurs when the gum tissues that partially cover an erupted tooth become inflamed and edematous [57]. The main causes of occurrence of this pathology are poor oral hygiene, accumulation of food debris and bacteria in the pocket created between the tooth and gum, inflammation of soft tissues around partially erupted teeth, as well as local trauma. Without proper treatment, pericoronitis can progress to a localized bacterial infection (abscess or maxillary cellulitis) or even to a generalized infection (sepsis) [58].

Tooth decay is a major oral health problem in both children and adults. Dental caries result from the action of bacteria that generate acid causing the demineralization of hard dental tissue. The bacterial complex involved in tooth decay formation is vast and diverse, but several main types of bacteria play key roles in this process (*S. mutans*, *S. sobrinus*, *Lactobacillus*, *Actinomyces*) [59]. Bacteria feed on the sugars and carbohydrates consumed, producing acids that erode tooth enamel and lead to cavities. Bacteria attach to the surface of teeth forming a biofilm known as dental plaque and sugars in the diet, producing acids as a byproduct. Acids produced by bacteria begin to erode tooth enamel, leading to demineralization and eventually the formation of cavities. We can reduce these effects by having more calcium, phosphate, and fluoride in the saliva. Thus, saliva enriched with fluoride, calcium, and phosphate can reduce the effect of bacteria contributing to remineralization [60].

## 3. Controlled Releasing Systems Used in the Oral Cavity

### 3.1. Fast-Dissolving Oral Films (FDOF)

Fast-dissolving oral films have the advantage that they release the pharmaceutically active form of the drug in less than 1 min after placement in the oral cavity without the need for water or mastication. They can be used for systemic delivery through the sublingual and oral mucosa or for local action. They are suitable for patients experiencing nausea, vomiting, mouth ulcers, allergic conditions, and CNS disorders. The composition of fast-dissolving films includes polymers, active pharmaceutical substances, film stabilizers, plasticizers, dyes, surfactants, and saliva-stimulating agents [61,62,63]. Several drugs are already incorporated into fast-dissolving films [63]:

Antihistaminic (loratadine, cetirizine, azacitidine maleat, chlorpheniramine);

Analgezice (ketoprofen);

Antimigraines (sumatriptan succinate, zolmitriptan);

Antidiareice (loperamide);

Proton pump inhibitors (omeprazole).

An application in dentistry of fast-dissolving films is the obtaining of sux formulations containing chlorhexidine by the solvent casting method. After in vitro decay analyses, antimicrobial activity tracking and stability studies, as well as using infrared spectroscopy with Fourier modifications, it was concluded that chlorhexidine films offer promising results in the management of oral diseases [64,65,66,67].

### 3.2. Intraoral Mucoadhesive Systems

The oral mucosa, by its large surface area, is an excellent interface for mucoadhesive systems (tablets, patches, films, gels, wafers, gums) that can be applied either on the oral mucosa, the sublingual mucosa, or even on the gum. The most commonly used form refers to adhesive tablets (e.g., intraoral metronidazole tablets) [30,68,69].

Mucoadhesive tablets are used in oral cavity treatments and for systemic effects. For mucoadhesive action, a series of polymers are used, testing their different proportions (60% carbomer and 40% hydroxypropylmethylcellulose) [69,70]. In 2017, mucoadhesive tablets containing miconazole were obtained for the treatment of oropharyngeal candidiasis polymers of chitosan and hydoxypropylmethylcellulose as the mucoadhesive [70].

Mucoadhesive microspheres (microcapsules) are oral controlled releasing systems of particular importance. These systems are characterized by increased bioavailability as well as sustained and controlled drug delivery. Using bioadhesive polymers such as sodium alginate and chitosan, mucoadhesive microcapsules were obtained incorporating various antibiotics such as ciprofloxacin [71], minocycline [72], and tetracycline [26].

Double-layer systems contain a waterproof layer and a mucoadhesive layer so that the tablet has prolonged retention [73,74]. At the level of the oral cavity, the therapy especially targets oral candidiasis, gingivitis, dental caries, oral lesions, xerostomia, and oral carcinomas. Current research aims to obtain improved treatments for local pathology, as well as better yield and bioavailability [73,74].

Hydrogels, unlike tablets, have certain characteristic physicochemical properties such as tissue similarity, high proportion of water, and biocompatibility. These properties allow the drug to be administered as semisolid systems that are slowly encapsulated and released by channel diffusion in the hydrogel network and polymer matrix degradation [73,74,75]. Given that hydrogels show poor retention, improvement was achieved by adding polymers to the formulation. To increase the bioavailability of hydrogels, pH-sensitive hydrogels, thermoreversible hydrogels, and adherent hydrogels were produced [76,77].

Abruzzo et al. obtained an intraoral mucoadhesive film based on *Lactobacillus brevis*. The film proved to have very good mucoadhesion and a long release period of viable lactobacilli either at the level of a lesion at the oral mucosa or at the level of the entire oral cavity [78]. To achieve better discharge of active substances, mucoadhesive systems have been improved with solid dispersion of nanoparticles, microemulsions, and liposomes [79,80,81,82,83].

Composite wafers have good mucoadhesion, inflation index, and high load capacity [18,20,84,85]. Newer concepts in intraoral drug administration include microneedles (MN) and microcontainers (MCs), types of devices that have numerous advantages [19,86,87].

Microneedles are used for both diagnostic and therapeutic purposes, providing the active substance in a permanent and minimally invasive way, increasing bioavailability and reducing needle phobia. Microneedles are useful both in the faster delivery of macromolecules (insulin, growth hormone) [88] (Table 1), in intraoral DNA sampling, oral health monitoring, local anesthesia, and in early diagnosis of oral diseases. The results of clinical trials on insulin, vasopressin, and the parathyroid hormone led to the approval of oral formulations based on a glucagon-like peptide that proved useful in the treatment of type 2 diabetes [71]. There are different types of microneedles: solid, coated, dissolvable, or hollow, where choosing a specific option depends on the medicine to be released, as well as the type of tissue to which it is addressed. Microneedles have also been shown to be useful in delaying the absorption and lymphatic transit of toxins in snake/viper bites in combination with the use of a pressure bandage [3,87,88].

Microcontainers (MCs) play an important role in the delivery of medicines. Birk et al. obtained antibiotic micro-containers to treat *Pseudomonas aeruginosa* infections in oral biofilms [89]. Moreover, the use of microcontainers in topical treatment may lead to a reduction in required doses and the risk of developing resistance [90,91,92].

Chewing gum is a more recent form of transmucosal failure of medicinal substances. It has a higher potential for controlled failure than mucoadhesive tablets, also offering a longer yield interval with very few associated side effects. Chewing gums are an excellent carrier category of various medicinal substances useful for oral health [21]. Chewing gums can be used for both their local and systemic action. Domperidone maleate, a medicinal chewing gum with antiemetic action, shows a release ratio of 97% after 15 min of chewing [93].

IntelliDrugs are intraoral drugs whose release of the active substance is controlled by electronic devices with tiny digital systems [94,95]. So far, these galantamine-laden systems have been used for dementia patients, offering the possibility of rhythmic therapeutic dose failure, especially for patients with swallowing difficulties [94,95,96]. Although IntelliDrugs still require prototype optimization processes, it cannot be denied that they represent innovative systems in intraoral drug administration (Table 1).

**Table 1:** Oral advanced drug delivery systems.

Failure System	Place of Action	Active Substance	Release Principle of the Active Substance	Local (LE)/Systemic (SE) Effect	References
Iontophoretic patches	Oral cavity	Lidocaine, prilocaine, chlorhexidine dexamethasone	Transmucosal absorption from patch having 3 layers (mucoadhesive, wrapping, and release)	LE	do Coutoet al., 2021 [12], Ren et al., 2016 [3]
Microneedles	Oral cavity	Human insulin	Macromolecule releasing	SE	González-Moles et al., 2021 [88]
Microneedles	Oral cavity	Human growth hormone	Macromolecule releasing	SE	González-Moles et al., 2021 [88]
Microcapsules	Oral cavity	Antibiotics, ciprofloxacin	Disperse in auxiliary substance	LE/SE	Drucker et al., 2020 [71]
Microcapsules	Root canal	Minocycline	Microcapsules (ionic gelling) having as polymers alginate or chitosan	LE	Duque et al., 2019 [72]
Microcapsules	Periodontal regeneration defects	Tetracycline + lovastatin	Microcapsules (based on chitosan)	LE/SE	Lee et al., 2016 [26]
Microcapsules	Post-surgical dental pain	Naproxen	Capsules with submicron particles (phase 2 study)	LE	Weisman et al., 2021 [97]
Microparticles with semisolid formulation	Subgingival in paradontal pouch	Propolis	Spray drying (yielding in about 7 days), polymer used gelatin	LE	Sahu et al., 2023 [98]
Mucoadhesive gels	Oral mucosa	Curcumin	Mucoadhesive polymer platforms useful in precancerous lesions	LE	Agarwal et al., 2015 [99]
Mucoadhesive patches with botanical extracts named Perio-patch	Oral mucosa	Herbal extract	Transmucosal absorption	LE	Neagu et al., 2023 [100]
IntelliDrugs	Teeth	Naltrexone, codeine, diazepam	Drug reservoir	LE	Handler et al., 2019 [101]
Mucoadhesives	Whole oral cavity	Domperidone maleate	Gum	SE	Lopes et al., 2015 [93]

## 4. Potential Optimizations of Nanoplatforms with Intra- and Extraoral Use 

The proposed optimizations focus on innovative approaches to improve the administration of drugs used in oral pathology, especially through transmucosal and transdermal pathways, with local or systemic effects. Our main proposals refer to the use of microneedle saw systems (they are very well suited for use in dentistry, especially in areas with weaker vascularization, offering a larger contact surface and better yield of active substances), nano-designed mucoadhesive patches, liposomal creams, and copolymer membranes.

Although the antibacterial and antifungal action of essential oils is proven, there are few studies on the incorporation of essential oils into various controlled released systems used in the oral cavity. Our studies on the action of essential oils have been a concern since 2014 when we studied the antibacterial and antifungal action of geranium essential oil (obtained by distillation of the vegetative parts of the species *Pelargonium roseum*) on Gram-negative bacteria (*Pseudomonas aeruginosa*, *Proteus mirabilis*, *Escherichia coli*), Gram-positive bacteria (*Staphylococcus aureus*, *Escherichia coli*), and fungi (*Candida albicans*). We also compared the inhibition zones generated by geranium oil with the inhibition zones given by references to the antibacterial substances diffusion-metric method: fluoroquinolones (ciprofloxacin), sulfonamides (co-trimoxazole, sulfamethoxazole + trimetroprim), penicillins (ampicillin), cephalosporins (cephalotin, cefotoxim), aminoglycosides (gentamicin and amikacin). The results indicated an inhibition comparable to antibiotics in the case of *Pseudomonas aeruginosa* and *Staphylococcus aureus*, as well as a complete inhibition in the development of *Candida albicans* [102].

The first study identified by us, conducted by Hosny et al., refers to a nanoemulsion with applicability in herpes labialis, which combines the antiviral penciclovir with lavender oil with anesthetic action. Comparing its action with other penciclovir gels of the same concentration, it was found that optimized nanoemulsion with lavender oil provides a more efficient and sustained release to the oral mucosa [103].

The second study, elaborated by Muresan et al., whose research is on periodontitis, refers to the in vitro comparison of three dental hydrogels: oregano essential oil, Frankincense oil (frankincense), as well as the Thieves mixture including Frankincense oil and extracts of cloves, lemon, eucalyptus, and rosemary. To test the cytotoxicity of hydrogels, mesenchymal stem cells from the dental papillae of germs of human wisdom teeth were used. The study demonstrates that hydrogels enriched with essential oils exhibit antimicrobial action in vitro, especially for *Staphylococcus aureus* and *Bacillus cereus* [104].

Given the penetration enhancer qualities and intrinsic oral health benefits of essential oils [15,105], we propose their use in various controlled release systems for oral pathology. Taking into account these aspects, we consider that the study of the in vitro efficacy of preparations with controlled yield based on essential oils requires special attention from specialists in the field, especially about the cytotoxicity of products, and to the extent that it proves high efficiency and low cytotoxicity, in vivo studies should be initiated for these innovative pharmaceutical forms.

### 4.1. Optimization of Transdermal Systems in Neuropathic/Neuralgic Pain

Capsaicin adhesive dermal patches applied directly to the painful area provide pain relief for 12 weeks in patients with painful diabetic neuropathy and patients with HIV-associated neuropathy [22,23]. In neuropathic pain, both transdermal patches with lidocaine [23] and a combination of lidocaine and capsaicin have been used to reduce pain intensity, which has proven very effective in patients with cancerous neuropathic pain refractory to opioid treatment [106]. Lidocaine acts on sodium channels, inhibiting them. In trigeminal neuralgia and neuropathies, it is very beneficial to apply a thin layer of anesthetic applied intraorally. To be absorbed transmucosally, lidocaine must be mixed with methylcellulose [23,106].

Topical therapy with 20% benzocaine, capsaicin, serotonin and norepinephrine reuptake inhibitors, and tricyclic antidepressants may be involved in transdermal release [24]. In addition to lidocaine, which has been used in transdermal patches for trigeminal neuralgia, transdermal opioid patches (buprenorphine and fentanyl) are also used. Fentanyl patches yield 12.5, 25, 50, and 100 μg/h. The onset of effect of this patch is at 12 h and the analgesic action persists up to 72 h. Fentanyl is 90% biotransformed in the liver by P-450 CYP3A4 to the inactive metabolite norfentanil. The other component (10%) is not metabolized [24,107].

Postoperatively, fentanyl is not used in patch form due to the late onset of its effect, and fentanyl administration with micropump administration (IONSYS) is preferred, this system allows for accelerating the absorption of opioids by iontophoresis [107].

**Figure 2:**
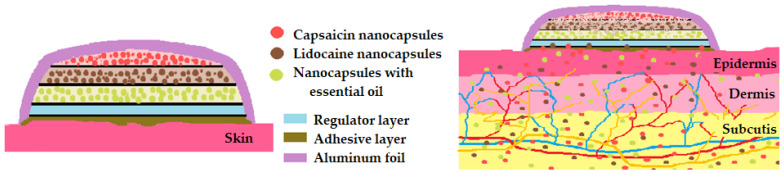
Optimization of transdermal patches in neuropathy/neuralgia.

In neuralgia and neuropathy, which are diseases of the neural path, we propose an optimization system such as the trilayer transdermal patch loaded with lidocaine, capsaicin, and lavender oil (due to the special anesthetic action of this essential oil [108]). Capsaicin transdermal patches reduce pain for 6–8 h; capsaicin binds to pain receptors and causes a burning sensation, so the association with lidocaine within this transdermal system reduces this discomfort [109]. On the other hand, lavender essential oil is helpful in neuropathic pain after 2–4 weeks of daily massage [110]. This oil is distinguished by its analgesic action given by one of its most important phytoconstituents, borneol [111]. Last, but not least, lavender oil is useful for the calming action of the burning sensation generated by capsaicin (Figure 2) [110,111].

### 4.2. Optimization of Transdermal Systems in Temporomandibular Joint Osteoarthritis

The most used treatment option is based on direct injection into the joint cavity, acting at the level of immune cells, as well as fibroblasts. Controlled failure systems are needed to prolong drug persistence and reduce the many side effects that solutions for injection in much higher doses can give [112]. Naproxen is a non-steroidal anti-inflammatory drug, which is used in the treatment of patients with osteoarthritis of the temporomandibular joint. In this regard, lipid systems of nanoparticles have been developed with high permeability, high efficacy, and low toxicity [112].

Polylactic-co-glycolic acid (PLGA) has a good capacity to encapsulate and form a film, which is why it is used to obtain innovative pharmaceutical products. Thus, in 2018, Ding et al. obtained PLGA microparticles and tracked in vivo the biocompatibility of these products. Intraarticular injections resulted in nociception for 6 days [113].

Parecoxib, another nonsteroidal anti-inflammatory that selectively acts on COX-2 and causes inhibition of prostaglandins, is used in chronic inflammation in osteoarthritis. In 2021, hyaluronic acid–PLGA microspheres loaded with this anti-inflammatory drug were obtained. A good parecoxib loading rate was achieved, and sustained release lasted up to 28 days [114].

For the treatment of osteoarthritis of the temporomandibular joint, intra-articular injections with chitosan can also be used. Chitosan is an amino polysaccharide structurally very similar to glycosaminoglycans that also possesses its own antibacterial, antifungal, anti-inflammatory, and analgesic action [31]. However, studies conducted by Li et al. [32] found that intraarticular administration of chitosan injections is uncomplicated (pain, inflammation in the temporomandibular joint area) but is not as effective as injection with autologous platelet-rich plasma (PRP) [31,32].

Another variant of treatment of temporomandibular joint osteoarthritis refers to the use of mesoporous silica nanoparticle systems. MSNs are real propellants for many drugs. MSN-CCs, having a cone structure, can distribute high-molecular-weight proteins. Thus, these systems were loaded with hyaluronan synthase 2, which is delivered to the synoviocytes of the temporomandibular joint. Single-dose administration of MSN-CC injections loaded with hyaluronan synthase 2 resulted in synovial inhibition for at least 3 weeks in rat studies [115].

**Figure 3:**
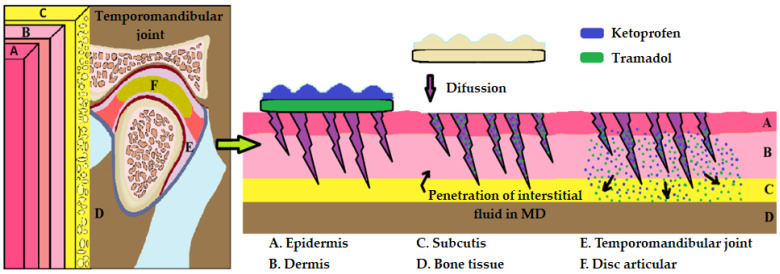
Optimization of the transdermal delivery system of drugs in osteoarthritis of the temporomandibular joint.

Microneedle patches (MNs) have sharp tips capable of painlessly piercing the stratum corneum and releasing drugs safely and effectively. Welds using tramadol-coated microneedles demonstrated greater efficacy than intra-articular injection of tramadol [33]. In these conditions, however, the release of tramadol is uncontrolled, mainly at the beginning of administration, which is a disadvantage. From this point of view, we believe that microneedles should be attached to nanoparticles with controlled release, which would greatly improve the efficiency and duration of the therapeutic effect. The first such system with saw needles was proposed in the case of osteoarthritis of the temporomandibular joint, where we considered that we could alternate this type of needle with some that have different heights, delivering the active substances at different levels of the dermis, with local effect, providing better penetration. We imagined this system of unevenly sized microneedles to which we attached bilayered patches, the first layer containing tramadol and the second layer having tanks loaded with ketoprofen nanoparticles (Figure 3).

Another optimization option that is suitable for osteoarthritis of the temporomandibular joint refers to the use of liposomal creams based on nonsteroidal anti-inflammatory drugs (diclofenac, ibuprofen, ketoprofen, naproxen) to which we associate essential oils for a synergistic action [15,105]. Among the essential oils, we prefer thymus, geranium, eucalyptus, and clove oils, whose anti-inflammatory action is already scientifically proven [105,116,117,118]. 

Liposome-based creams are ideal pharmaceutical forms of delivery of anti-inflammatories because they act in a targeted manner on the inflammatory focus, and demonstrate increased efficiency concerning systemic forms, with minimal side effects. Liposomes can play the role of solubility matrix, local deposits, or even penetration amplifiers [34]. To increase skin permeability a series of physical amplifiers (microneedles, iontophoresis, sonophoresis, and electrophoresis) may be used [31,32,33,34,115]. 

**Figure 4:**
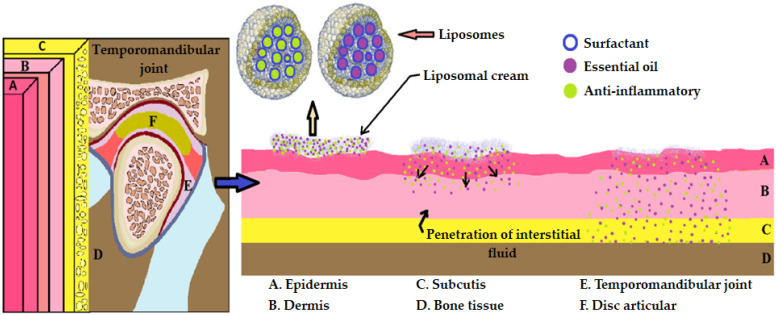
Optimization of liposomal cream in osteoarthritis of the temporomandibular joint.

The vast majority of existing studies to date related to the incorporation of certain NSAIDs such as liposomes or niosomes are preclinical studies in animals, aimed primarily at an efficacy and safety profile [119]. On the phytochemical side, there are several such studies, but we have not found a formulation associating NSAIDs with essential oils with intrinsic anti-inflammatory activity (Figure 4).

### 4.3. Optimization of Transmucosal Controlled Failure Systems in Oral Cancer

Most anticancer drugs cannot be taken orally because of their low aqueous solubility. However, when incorporated into the appropriate biomaterials, controlled release forms with local or systemic actions and increased efficacy can be obtained. We will now present the drugs most commonly used in the treatment of various forms of oral cancers and targeted release systems for which clinical trials are being conducted [53,120].

Cetuximab is a monoclonal antibody used to treat patients with oral cancer. To reduce severe adverse effects and increase the reactivity of cetuximab, it has been combined with gold nanoparticles and radiotherapy procedures. Gold nanoparticles increased the efficiency of cetuximab, but complete eradication of tumors in vivo was not observed [121].

Cisplatin, a chemotherapy agent that causes apoptosis of cancer cells with the ability to bind purine bases in DNA structure, is used in oral cancer in the form of nano-designed cisplatin patches for which phase I/II clinical trials have been conducted. This formulation leads to both an increase in the effectiveness of the active substance and a reduction in its toxicity [27].

To reduce nephrotoxicity and neurotoxicity, cisplatin-loaded nanoparticles were also obtained with a higher efficacy than oral cisplatin in solution. To achieve the highest loading efficiency of the drug possible, the temperature and concentration of polybutylcyanoacrylate were raised [122].

In 2023, Habib et al. developed mucoadhesive patches loaded with cryogenic liposomes of doxorubicin, an anticancer agent. The alginate/liposome ratio influences the degree of adhesion of the patch to the tongue as well as the release profile of liposomes from the matrix. The effect of these patches has been demonstrated in vivo in mice with squamous cancer tumors. Compared to oral administration or even implanted patches, mucoadhesive patches applied directly to the lesion are more effective and free of side effects [123].

In the case of docetaxel delivered in polymeric nanocapsules, a scaffold was first obtained by grafting folic acid and thiol groups to chitosan. Then, silver nanoclusters were synthesized in situ and core-coating nanocapsules were obtained with docetaxel in the core and silver nanocapsules distributed in the shell. A nine-fold increase in bioavailability and 6.8-fold half-life were observed. Studies on changes in biochemical and histopathological parameters at renal and hepatic levels did not reveal high toxicity in the mouse group [124,125,126].

The use of nanotechnology in cancer therapy increases administration efficiency, decreases systemic toxicity, and last, but not least, decreases treatment costs. Certain nanoparticles such as superparamagnetic iron oxide or carbon nanotubes can even cause cancers in some animals. The DDS most commonly used are lipid nanoparticles or polymers as obtained in the case of nivolumab (Table 2) [28,29,127,128,129].

**Figure 5:**
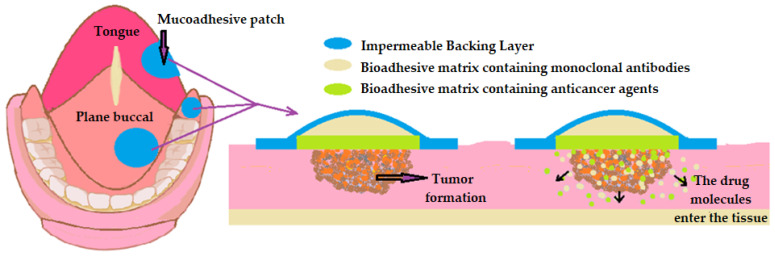
Optimizing the release of anticancer agents using nano-designed mucoadhesive patches with doxorubicin and cetuximab.

In oral cancer, we propose as a form of controlled failure the use of nano-designed adhesive patches for the local effect, on the one hand, and for the ease of their application and removal, on the other hand. We believe that microneedles are not suitable for cancer therapy due to the increased potential for metastasis. Mucoadhesive patches should combine anticancer agents with local and systemic effects such as cisplatin or doxorubicin with monoclonal antibodies such as cetuximab. We believe that microneedles are not an option in cancer, as they can cause dispersal of cancer cells. The principle of releasing medicinal substances imagined by us is shown in (Figure 5).

### 4.4. Optimization of Transmucosal Controlled Release Systems in Periodontal Pockets 

Microneedles can play an important role in periodontal surgery, accelerating healing processes. The release of platelet-derived growth factor (PGF) as well as TGF-α and TGF-ß (transforming growth factors α and ß) and fibroblast growth factor, as a result of minimal bleeding produced during application, help in the healing process. All needles influence new collagen formation and neovascularization through fibroblast proliferation and migration [130,131,132,133,134].

**Figure 6:**
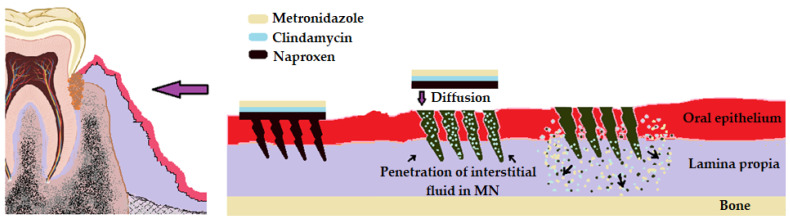
Optimization of controlled release systems in periodontal pockets.

In the therapy of periodontal pockets, we propose a system for optimizing microneedles, as well as the use of saw needles that are very suitable in dentistry due to the larger contact surface created, the fact that the vascularization is not very good in this area, and the fact that this microneedle system allows a better dispersion of the drug. We propose, to obtain better absorption, the use of nanocarriers associated with the MN-polymer system to obtain a synergistic local effect. As a pharmacological variant, we propose the association of chemotherapy and an antibiotic of the lincosamide type, more precisely metronidazole with clindamycin, which, from experience, gives excellent results in the treatment of periodontal pockets, adhering to both the infectious process and the inflammatory process. To reduce associated inflammation, we propose adding to this formula a nonsteroidal anti-inflammatory such as naproxen (Figure 6).

### 4.5. Optimization of Transmucosal Controlled Release Systems in Pericoronitis 

Consulting the literature related to innovative treatments based on transdermal systems used in pericoronitis, we found that there are no targeted studies for this condition. However, Todorovic et al. propose a transdermal system for postoperative pain management after wisdom teeth extraction. Their study involves the use of a fentanyl patch applied postoperatively for which the degree of pain perception on the postoperative VAS (Visual Analogue Scale) was monitored [135].

**Figure 7:**
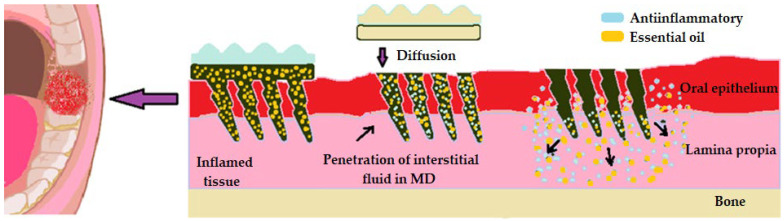
Optimization of microneedles used in the treatment of pericoronitis.

In pericoronitis, where managing inflammation and acute pain are the main problems to solve, we advocate for saw needle systems. Our optimization variant for obtaining a local effect envisages the dispersion, on the one hand, of microneedles loaded with nanoparticles of essential oils, and on the other hand, a tank-type system loaded with nonsteroidal anti-inflammatory drugs, which is so useful in this type of gingival inflammation. From the multitude of essential oils available to us, we especially appreciate the essential oils of thymus, eucalyptus, geranium, and cloves, which are distinguished by their anti-inflammatory, antibacterial, and analgesic action. All these oils also have a remarkable antiseptic action, which is useful in maintaining oral health (Figure 7) [136,137].

### 4.6. Optimization of Copolymer Membranes Loaded with Fluorine Nanoparticles and Essential Oils in Maintaining Oral Health (Dental Caries, Lesions in the Oral Cavity) 

In 2019, Tatsi et al. developed a controlled fluoride release system in a pilot study that demonstrated an increase in fluoride value from 0.02 to 0.06 ppm in unstimulated human saliva [138].

**Figure 8:**
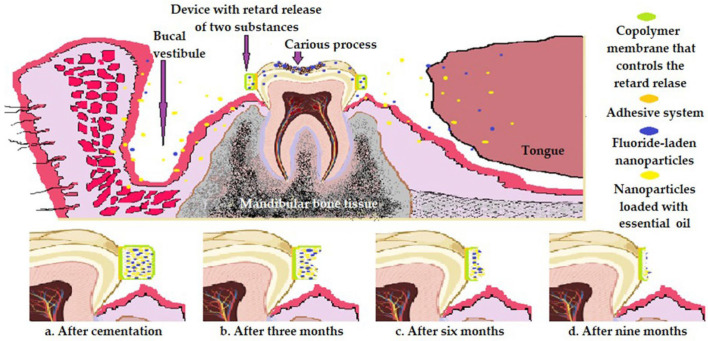
Optimization of copolymer membranes loaded with fluorine nanoparticles and essential oils.

The system proposed by us refers to a copolymer membrane containing fluorine nanoparticles and essential oils (Figure 8). This copolymer membrane will be cemented directly on the human tooth to give up the two components (fluorine and essential oil) at the level of the entire oral cavity, and essential oils have antibacterial, antiseptic, antiviral, anti-inflammatory, and slightly anesthetic effects that vary in intensity from one type to another [139]. We believe that we should pay more attention to microcapsules based on essential oils and their application in dentistry either on their own or associated with MN-polymers. We must bear in mind that essential oils are absorbed in the small intestine in the proximal area, and excess release in the stomach can generate nausea, tachycardia, dizziness, etc. [140]. Thus, to control release, microencapsulation seems to be an excellent option. Given that antibacterial treatments generate several side effects, we believe that essential oils are an excellent alternative when a substitute to antibiotic therapy is required. Essential oils are distinguished by their antimicrobial, antifungal, anti-inflammatory, anticancer, and healing effects, but their delivery to specific receptors is a challenge due to their physicochemical properties: low solubility in water and high volatility [139,140]. To this end, nanotechnology is the only sustained and controlled strategy for yielding essential oils [139,140]. An important aspect to mention is related to the fact that, in addition to intrinsic benefits for oral health, they play a role as penetration enhancers [15,96]. However, further studies into their efficacy and safety are needed.

The optimization system proposed by us regarding the copolymer membrane includes fluorine and essential oil nanoparticles, but also the other optimization systems: patches with microneedles in the saw with alternating heights, liposomal cream based on nonsteroidal anti-inflammatory drugs and essential oils (thymus, geranium, eucalyptus, cloves), nano-designed mucoadhesive patches, nanocarriers associated with microneedle systems, microneedle saw system loaded with essential oil nanoparticles (to which we attach tanks with nonsteroidal anti-inflammatory drugs), and copolymer membranes loaded with fluorine nanoparticles and essential oils, as described in a synthesis in Table 3.

## 5. Conclusions

Although it is one of the most used routes of administration, the oral route has been proven in certain situations (fever, vomiting, diarrhea, unconsciousness, psychiatric patients) to be unsuitable for both adults and children.

If these classic formulations pose problems, we can resort to innovative formulation strategies. To increase the bioavailability, permeability, and solubility of the drug, nanocarriers embedded in nano-drug delivery systems (NN-DS) can be used.

Following the development of nanotechnology, oral administration systems have been developed based on natural polymers (dextran, chitosan, alginate, gelatin) or synthetic polymers (polycaprolactone (PCL), polyglycolide, polycyanoacrylate). All these techniques are based on reducing particle size and decreasing the rate of dissolution.

To obtain better aqueous solubility of drugs, several factors must be taken into account, such as particle size, selection of the most suitable salts, a good choice of surfactant, micro/nanonization, amorphization, crystal engineering, etc. Surfactants increase bioavailability through multiple mechanisms, generating both the solubility and permeability of drugs. However, their use in high concentrations raises safety concerns. Also, the transformation of certain acids or bases into salts increases solubility, as well as causes changes in pH.

At the level of the oral cavity, the latest pharmaceutical forms called smart intelligent systems (SIS) offer several advantages: they penetrate the oral mucosa barrier, have good adhesion, are not easily swallowed, and offer a controlled release and high bioavailability. The fact that these pharmaceutical forms allow a controlled release in therapeutic doses for a long period while minimizing side effects makes them suitable for use in oral pathology.

## Figures and Tables

**Table 2:** Oral advanced drug delivery systems in oral cancer.

Nr crt.	Active Substance	Pharmacological Group	Yielding Systems/Carriers	Improvement	Local (LE)/Systemic (SE) Effect	References
1.	Cetuximab	Monoclonal antibodies	Gold nanoparticles	Increased efficacy of the active substance	SE	Sato et al., 2023 [28]
	Cetuximab + cisplatin	Monoclonal antibodies + chemotherapeutic agent	Gold nanoparticles	Used in aggressive forms of metastatic cancer	SE	Sürer et al., 2021 [127]
2.	Cisplatina	Chemotherapy agent	Nano-designed cisplatin patch	Used in aggressive forms of metastatic cancer	SE	Goldberg et al., 2022 [27]
	Cisplatina		Polybutylcyanoacrylate (PEG) nanoparticles	Improve efficacy, reduce toxicity	SE	Alavi et al., 2019 [122]
3.	Doxorubicin	Powerful anticancer agent	Mucoadhesive patches loaded with liposomes	Increased efficiency and reduced side effects	LE/SE	Habib et al., 2023 [123]
4.	Docetaxel	Anticancer agent	Polymeric nanocapsules containing silver nanoclusters	Obtaining a much-increased bioavailability and half-life	LE/SE	Sohail et al., 2018 [124]
5.	Nivolumab	Monoclonal antibodies	Polymer/lipid nanoparticles	May decrease immunosuppression and promote active substances at the site of action	SE	Hanna et al. 2024 [125], Wang et al., 2021 [126]

**Table 3:** Synthesis of optimization of drug delivery systems in various oral pathologies.

Nr crt.	Oral Pathologies	Proposed Yield Systems	Local (LE)/Systemic (SE) Effect	Optimization	References
1.	Neuropathy/neuralgia	Transdermal patches with lidocaine, capsaicin, and lavender oil	LE	Lavender oil as penetration enhancer/synergistic action of three pharmacological principles with different mechanisms of action	Pickering et al., 2020 [22], Tsai et al., 2023 [23], Zhao et al., 2021 [24]
2. A	Osteoarthritis of the temporomandibular joint	Plasters with micro-needle saws with alternating heights	LE	Saw-type needles and their alternation with penetration on different height levels	Derwich et al., 2022 [31], Cigerim et al., 2020 [112]
2. B	Osteoarthritis of the temporomandibular joint	Liposomal cream based on non-steroidal, anti-inflammatory, and essential oils (thymus, geranium, eucalyptus, cloves)	LE	Combining nanoparticles loaded with non-steroidal anti-inflammatory drugs with nanoparticles loaded with essential oils for a synergistic effect	Chen et al., 2015 [15], Hou et al., 2022 [105]
3.	Oral cancer	Nanoprojected mucoadhesive patches	LE/SE	Mucoadhesive patches are an excellent option in oral cancer and combining two therapeutic groups of anticancer agents and monoclonal antibodies gives us increased chances in the process of remission or even healing	Goldberg et al., 2022 [27], Sato et al., 2023 [28], Habib et al., 2023 [123]
4.	Periodontal pockets	Nanocarriers associated with micro-ace systems	LE/Prevent the dissemination of the infectious process	1. The use of needles in the saw taking into account the weak vascularized area 2. Conjugation of three drug groups: antibiotics, chemotherapy, and non-steroidal anti-inflammatory drugs	Bilal et al., 2021 [18], Yu et al., 2023 [131], Amato et al., 2023 [134]
5.	Pericoronitis	Micro-needle saw system loaded with nanoparticles of essential oils, and we attach tanks with non-steroidal anti-inflammatory	LE/Role in maintaining oral health through the action of essential oils	Use of dual-role essential oils: 1. Amplifier agent in nanoformulation 2. By the anti-inflammatory action, antibacterial, and soothing role in maintaining oral health	Aljaafari et al., 2021 [136], Kong et al., 2022 [137]
6.	Oral health/dental caries	Copolymer membranes loaded with fluorine nanoparticles and essential oils	LE/Role in maintaining oral health through the action of essential oils	Compared to the existing data, we propose this association of fluoride nanoparticles with essential oil nanoparticles with real oral health benefits	Jiao et al., 2019 [8], Liang et al., 2020 [9], Tatsi et al., 2019 [138], Albuquerque et al., 2022 [139]

## Data Availability

Not applicable.
